# Anti-aging activities of extracts from Tunisian medicinal halophytes and their aromatic constituents

**DOI:** 10.17179/excli2017-244

**Published:** 2017-05-22

**Authors:** A. Jdey, H. Falleh, S. Ben Jannet, K. Mkadmini Hammi, X. Dauvergne, C. Magné, R. Ksouri

**Affiliations:** 1 Faculty of Sciences at Bizerte, 7021 Zarzouna, Tunisia; 2 Laboratory of Aromatic and Medicinal Plants, Centre for Biotechnologies, Technopôle of Borj-Cédria, BP 901, 2050 Hammam-Lif, Tunisia; 3 EA 2219 Geoarchitecture, University of Western Brittany, 6 av. V. Le Gorgeu, CS 93837, Brest Cedex 3, France

**Keywords:** anti-aging, aromatic composition, biological activities, ethnobotany, medicinal halophytes

## Abstract

Six medicinal halophytes widely represented in North Africa and commonly used in traditional medicine were screened for pharmacological properties to set out new promising sources of natural ingredients for cosmetic or nutraceutical applications. Thus, *Citrullus colocynthis*, *Cleome arabica*, *Daemia cordata*,* Haloxylon articulatum*, *Pituranthos scoparius* and *Scorzonera undulata* were examined for their *in vitro* antioxidant (DPPH scavenging and superoxide anion-scavenging, *β*-carotene bleaching inhibition and iron-reducing tests), antibacterial (microdilution method, against four human pathogenic bacteria) and anti-tyrosinase activities. Besides, their aromatic composition was determined by RP-HPLC. *H. articulatum* shoot extracts exhibited the strongest antioxidant activity and inhibited efficiently the growth of *Salmonella enterica *and *Escherichia coli*. *P. scoparius* and *C. arabica* inhibited slightly monophenolase, whereas *H. articulatum* was the most efficient inhibitor of diphenolase activity. Furthermore, *H. articulatum* exhibited the highest aromatic content (3.4 % DW), with dopamine as the major compound. These observations suggest that shoot extract of *H. articulatum*, and to a lesser extent of *C. arabica*, could be used as antioxidant, antibiotic as well as new natural skin lightening agents. Also, possible implication of aromatic compounds in anti-tyrosinase activity is discussed.

## Introduction

Consumers are currently demanding less use of chemicals or minimally processed plant-derived food, so more attention had been paid to search for naturally occurring substances to treat illnesses or for health care. This is particularly true for plant materials that act as alternative antimicrobial or antioxidant sources. Therefore, the antioxidant phytochemicals from vegetables, fruits and medicinal plants have received increasing concern in the last decades for their potential role in preventing human diseases. Besides, more attention is being paid to the use of natural plant extracts in the cosmetic industry (Rangkadilok et al., 2007[42]; Shen at al., 2010[47]). Here, the use of natural products as antioxidant, antimicrobial and anti-aging agents provides a better alternative to the wide and injudicious use of synthetic agents. 

Halophytes are salt-tolerant plants living in hostile natural environment characterized with a number of stressful conditions (*e.g.* drought, salinity, UV radiations, and extreme temperatures). In those conditions, they experience a strong and permanent oxidative stress and produce harmful ROS. As a consequence, they are constitutively equipped with a powerful antioxidant system that includes enzymatic and non-enzymatic components (Ksouri et al., 2012[27]; Falleh et al., 2013[17]). Among the latter, aromatic compounds are secondary metabolites that may participate in cell protection against the harmful action of xenobiotics or UV radiations through their capacity to quench reactive oxygen species (ROS) and to regulate oxidative pathways including melanogenesis (Rice-Evans et al., 1997[43]; Panich et al., 2010[38]). These properties make halophyte aromatic compounds of great potential for cosmetic and pharmaceutical uses. Accordingly, investigations have been addressed to identify novel and natural bioactive sources among medicinal halophytes, which could be used for skin-protective applications.

With the aim at setting out new natural sources of cosmetic interest, six medicinal halophytes were, for the first time, tested for their ability to inhibit tyrosinase, as well as for their antioxidant and antimicrobial capacities. Moreover, aromatic compounds were analyzed in these species, as possible bioactive compounds responsible for those activities.

## Materials and Methods

### 1. Plant material

Since traditional ethnomedicinal use of plant is recognized as an important potential source for compounds used in mainstream medicine, six halophytic species widely used in African folk medicine have been selected for scientific investigations: *Citrullus colocynthis* (L.) Schrad., *Cleome arabica *(L.), *Daemia cordata *(Forssk.) R. Br. ex Schult.,* Haloxylon articulatum* (Cav.) Bunge, *Pituranthos scoparius* (Coss. & Durieu) Benth. and *Scorzonera undulata* Vahl. Main characteristics of the selected plants are resumed in Table 1(Tab. 1).

Fruit of *Citrullus colocynthis* have long been used for the treatment of diabetes, cancers, microbial infection, ulcer, asthma, inflammation, jaundice, and urinary disease in Asian and African countries (Ziyyat et al., 1997[57]; Qureshi et al., 2010[40]; Rajamanickam et al., 2010[41]). Moreover, its dried fruit is a strong laxative and an abortifacient (Alkofahi et al., 1996[2]; Madari and Jabobs, 2004[30]), and it may be used against arthritis, parasitic worms and skin diseases. Is has been shown to contain colocynthin and vegetative organs colocynthetin, two bioactive glycosides. Roots contain elaterin, a poisonous cathartic substance used as a fat burner in Asian herbal, and the cytotoxic diterpenoid hentriacontane.

*Cleome arabica* has long been used as folk medicine in North Africa to treat scabies and inflammation (Ahmad et al., 1990[1]; Tsichritzis et al., 1993[52]) as well as rheumatic pains (Bouriche and Arnhold, 2010[7]). It also possesses antimicrobial (Takhi et al., 2011[50]), antioxidant (Alkofahi et al., 1996[2]; Selloum et al., 1997[45]), acricidal (Kemassi et al., 2012[24]), and cytotoxic activities (Nagaya et al., 1997[35]). 

*Daemia cordata* (=* Pergularia* *tomentosa, Telosma tomentosa*) leaves and roots are prepared as an infusion, decoction, or powder, and taken orally or applied externally on the skin. It is reputed for diverse folk uses as an antirheumatic, laxative, abortive or hepatoprotective agent. It is also efficient in treatment of some skin diseases or against asthma and bronchitis (Gohar et al., 2000[18]; Suresh Kumar and Mishra, 2008[49]). 

*Haloxylon articulatum *(= *Caroxylon articulatum,* *Salsola articulata, Hammada articulata***) **grows in sandy habitats in subtropical regions from North Africa to China. Although it has no interest as forage, it is important for soil conservation. Eight alkaloids (mainly isoquinolins and beta-carboline derivatives) have been reported from *H. articulatum* aerial parts (Benkrief et al., 1990[4]; El-Shazly and Wink, 2003[16]), and it has some traditional uses in Saharian folkloric medicine against scorpion stings, cooling, indigestion, and hypertension (Hammiche and Maiza, 2006[19]).

In traditional medicine, stems and leaves of *Pituranthos scoparius* have been used in the treatment of measles, fever, rheumatism, asthma, jaundice, digestive difficulties, urinary infections, diabetes, hepatitis and postpartum spasms or pains (Boukef, 1986[6]; Shaker et al., 1999[46]). They are also applied locally as a poultice against snake and scorpion bites (Boukef, 1986[6]; Hammiche and Maiza, 2006[19]).

*Scorzonera* species are used against pulmonary diseases, colds, for the treatment of wounds and gastro-intestinal disorders, as well as for their stomachic, diuretic, galactogogue, antipyretic and appetizing effects in European traditional medicine (Zidorn et al., 2003[56]; Tsevegsuren et al., 2007[51]).

### 2. Sampling and extraction

Aerial parts of the six xerohalophytic species were harvested in summer 2014 from Sidi-Bouzid. The site is located at the middle-east of Tunisia (latitude 35°2'24''N; longitude 9°30'0''E), in a region characterized with arid bioclimatic stage. All samples were rinsed with distilled water, freeze-dried and ground in a Mettler AE 200 blender. 

Extraction was performed by magnetic stirring of 2.5 g dry powder in 25 ml aqueous ethanol (50 %, v/v) for 16 h at 4 °C. Then, the mixture was filtered through a Whatman No 4 filter paper and evaporated under vacuum to dryness. The dry residue was resuspended in 50 % ethanol at a concentration of 100 mg/ml and stored at -27 °C until analyses.

### 3. Determination of antioxidant activities

#### 3.1. DPPH scavenging activity

The hydrogen atom or electron donation ability of the extracts was measured from the bleaching of purple colored methanol solution of 1,1-diphenyl-2picrylhydrazyl (DPPH) according to the method described by Sokmen et al. (2004[48]). One milliliter of various concentrations of the ethanol extracts was added to 250 µl of 0.2 mM DPPH radical solution in methanol. The mixtures were shaken vigorously and allowed to stand for 30 min in the dark. The absorbance of the resulting solutions was measured at 517 nm and butylated hydroxytoluene (BHT) was used as a positive control. Inhibition of DPPH radical was calculated as follows:

DPPH^•^ scavenging effect (%) = [100 * (A_0_ - A_1_) / A_0_] (1)

where A_0_ and A_1_ are the absorbance values of the control and of the sample at 30 min, respectively. The antiradical activity was expressed as IC_50_ (µg**^.^**ml^−1^), a low IC_50_ value corresponding to a high antioxidant activity. All samples were analyzed in triplicate.

#### 3.2. β-carotene bleaching test

A slightly modified method of that described by Koleva et al. (2002[26]) was employed to estimate shoot extract capacity to inhibit the *β*-carotene bleaching. Two milligrams of *β*-carotene were dissolved in 20 ml of chloroform, and to 4 ml of this solution linoleic acid (40 mg) and Tween 40 (400 mg) were added. Chloroform was evaporated under vacuum at 40 °C and 100 ml of oxygenated water were added, then the fresh emulsion was vigorously shaken. An aliquot (150 µl) of the *β*-carotene/linoleic acid emulsion was distributed in 96-well microtiter plates (NUNC microplate, Fisher Bioblock) and methanol solutions of the test samples or authentic standards (10 µl) were added. Three replicates were prepared for each concentration. The absorbance of all wells was measured at 470 nm using a microtiter reader (Multiskan EAR 400, Labsystems), both immediately (t = 0 min) and after 120 min of incubation at 50 °C. The antioxidant activity of the BHT standard and of plant extracts was calculated in percentage of *β*-carotene bleaching inhibition as follows:

% inhibition = (S − C_120 _/ C_0_ − C_120_) _*_ 100 (2)

where C_0_ and C_120_ are the absorbances of the control at 0 and 120 min, respectively, and S is the sample absorbance at 120 min. Results were expressed as IC_50_ values (µg**^.^**ml^−1^).

#### 3.3. Superoxide anion radical-scavenging activity 

Superoxide scavenging capacity was assessed according to Duh et al. (1999[14]). The reaction mixture contained 0.2 ml of shoot extracts at different concentrations, 0.2 ml of 60 mM PMS, 0.2 ml of 677 mM NADH and 0.2 ml of 144 mM NBT, all in phosphate buffer (0.1 M, pH 7.4). After 5 min of incubation at room temperature, the absorbance was read at 560 nm against blank. The inhibition percentage of superoxide anion generation was calculated using the previous formula (2). As for the antiradical activity, the antioxidant activity in shoot extracts was expressed as IC_50_ in µg**^.^**ml^-1^.

#### 3.4. Iron reducing power

The iron reducing power was determined based on the transformation of Fe^3+^ to Fe^2+^ induced by the plant extracts according to the method of Oyaizu (1986[37]). Sample solutions at different concentrations were prepared in 1 ml of 0.2 M phosphate buffer (pH 6.6) and mixed with 1 ml of potassium ferricyanide (1 %, w/v). Tube was incubated at 50 °C for 20 min. Afterwards, 1 ml of TCA (10 %) were added and the mixture was centrifuged for 10 min at 1000 *g*. Supernatant (1 ml) was mixed with distilled water (1 ml) and 0.1 ml of ferric chloride (0.1 %, w/v), and the absorbance was read at 700 nm. Higher absorbance values of the reaction mixture indicate a greater reducing power. EC_50_ value (µg**^.^**ml^−1^) is the effective concentration of the extract at which the absorbance was reduced by 50 % and it was obtained from linear regression analysis. All samples were analyzed in triplicate.

### 4. Evaluation of the antibacterial activity

#### 4.1. Microorganisms tested

Antibacterial activity was screened against four human pathogenic bacteria including the Gram positive *Micrococcus luteus *(ATCC 10240) and* Staphylococcus aureus subsp. aureus *(ATCC 33862), and the Gram negative *Escherichia coli *(ATCC 4157) and *Salmonella enterica subsp. arizonae *(ATCC 13314).

#### 4.2. Antibacterial bioassay

Strains were grown in liquid nutrient broth (Difco Surrey, England) at 37 °C for 24 h before being used. A microplate-bioassay (microdilution) was used to study the antimicrobial activities of plant extract. An aliquot of each extract, corresponding to 100 µg plant dry matter, was dropped in sterile 96-well plates. After complete evaporation of the solvent, 100 µl of microorganism suspensions (10^2^ cells**^.^**ml^−1^) obtained by dilution from the culture tube (10^8^ cells**^.^**ml^−1^) were added to each well. Microbial suspension was used alone as positive control or in the presence of antibiotic mixture (5 mg**^.^**ml^-1^ streptomycin and 10 mg**^.^**ml^−1^ penicillin G) as negative control. Then, the microplate was aseptically sealed, agitated and incubated at 30 °C for 24 h. Finally, microorganism growth was estimated by reading the absorbance in each well at 405 nm with a microplate spectrophotometer (Multiskan MCC/340, Titertek). Antibacterial activity was expressed both in percentage of growth inhibition and in microbial susceptibility index (MSI, proportion of extracts inhibiting one microorganism). The absorbance data allowed us to calculate the percentage of growth inhibition using the following formula:

Growth inhibition (%) = 100 − [100 _*_ (A_sample_-A_SC_) / (A_GC_-A_SC_)] (3)

where A_SC_ and A_GC_ are the absorbances of the sterility control (negative control) and of the growth control (positive control), respectively. Then, the microbial susceptibility index (MSI) was calculated using the following formula: 

MSI = 100 _*_ (No. of extracts inhibiting one strain / Total No. of extracts) (4)

Thus, MSI values for a particular strain range from '0' (resistant) to '100' (susceptible).

### 5. Evaluation of tyrosinase inhibition properties

Tyrosinase inhibition activity was determined as described by Momtaz et al. (2008[34]), with L-3,4-dihydroxyphenylalanine (L-DOPA) or L-tyrosine as substrates. Samples were dissolved in dimethyl sulfoxide (DMSO), and further diluted in potassium phosphate buffer (50 mM, pH 6.5). Assays were carried out in a 96-well microtiter plate and absorbances were red on a Multiskan FC microplate reader (Thermo scientific technologies, China). Each prepared sample (70 μl) was mixed with 30 μl of tyrosinase (333 Units**^.^**ml^-1^ in phosphate buffer, pH 6.5). After 5 min of incubation at room temperature, 110 μl of substrate (2 mM L-tyrosine or 12 mM L-DOPA) were added and the reaction mixture was incubated further for 30 min. Kojic acid was used as a positive control, as well as a blank containing all the components except L-tyrosine or L-DOPA. Absorbance was measured at 492 nm, and the percentage of tyrosinase inhibition was calculated as follows:

% inhibition = [(A_control_ - A_sample_) / A_control_] _*_ 100 (5)

where A_control_ and A_sample_ are the absorbances of the blank and of the test reaction mixture (containing extract or kojic acid), respectively. The IC_50_ values of extracts and kojic acid were calculated.

### 6. Aromatic compound determination

The separation of aromatic compounds from plant extracts was done using HPLC Agilent 1260 system (Agilent technologies, Germany) equipped with a reversed phase C18 analytical column of 4.6 x 100 mm, and 3.5 μm particle size (Zorbax Eclipse XDB C18). Diode array detector was set to a scanning range of 200-400 nm and column temperature was maintained at 25 °C. The injected volumes were 5 μl and the flow-rate of mobile phase was 0.4 ml**^.^**min^-1^. Mobile phase was constituted of a mixture of two solvents, methanol (A) and 0.1 % formic acid (B), with the following gradient program: 10 % A 90 % B (0-5 min), 20 % A 80 % B (5-10 min), 30 % A 70 % B (10-15 min), 50 % A 50 % B (15-20 min), 70 % A 30 % B (20-25 min), 90 % A 10 % B (25-30 min), 50 % A 50 % B (30-35 min) and 10 % A 90 % B (35-36 min). Chromatograms were monitored at 254 nm and peak identification was obtained by comparing the retention time and the UV spectra of each peak with those of pure standards (purity > 99%) purchased from Sigma (Table 2(Tab. 2)). Moreover, individual aromatic contents were obtained from calibration curve with standards. 

### 7. Statistical analyses

All extractions and assays were conducted in triplicate. The means were compared by using one-way analysis of variance (ANOVA) followed by Duncan's multiple range tests performed by the ''Statistica v 5.1'' software (Statsoft, 2008). The differences between individual means were deemed to be significant at *P *< 0.05. Moreover, a correlation study was performed between all measured parameters in order to show possible relationships between activities and aromatic contents.

## Results

### 1. Antioxidant activities of halophyte shoot extracts

**Figure 1:**
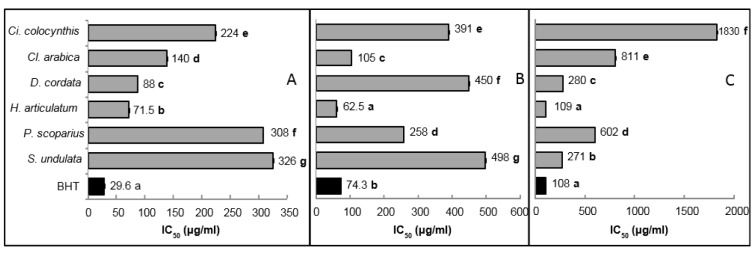
DPPH radical-scavenging (A), *β*-carotene bleaching inhibition (B) and superoxide anion radical-scavenging (C) activities of halophyte shoot extracts (50 % ethanol). All activities were expressed as IC_50_ (µg^.^ml^-1^). Means of three replicates followed by different letters are significantly different at *P *< 0.05.

The six studied halophytes differed significantly in their capacity to stabilize DPPH radical (Figure 1A(Fig. 1)). Of the six species, *H. articulatum* exhibited the highest antiradical activity, with the lowest IC_50 _value (71.5 µg**^.^**ml^-1^), followed by *D. cordata*. Conversely, *P. scoparius* and *S. undulata* showed a limited activity, as compared to the BHT standard.

The efficiency of the six halophytic extracts to inhibit the auto-oxidation of polyunsaturated fatty acids was evaluated using the *β*-carotene bleaching test. As shown in Figure 1B(Fig. 1), *H. articulatum *extract exhibited the highest inhibition (IC_50_ = 62.5 µg**^.^**ml^−1^), followed by *C. arabica*. The other four species showed only a moderate antioxidant activity using this bioassay, as compared to the standard BHT.

Results from the superoxide-scavenging test showed that the six species quench superoxide anion to significantly different extents (Figure 1C(Fig. 1)). Here again, *H. articulatum *extract exhibited the highest antiradical potential (IC_50_ = 109 µg**^.^**ml^−1^), representing a 2.5 fold higher activity than *D. cordata* and *S. undulata*. The other three species were almost inactive compared to BHT.

**Figure 2:**
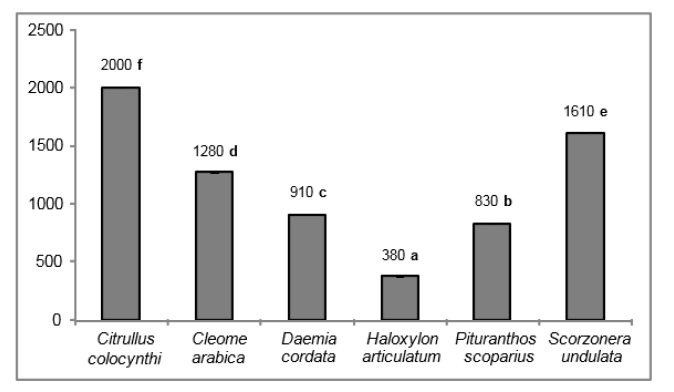
Iron reducing power, expressed as EC_50_ (µg^.^ml^-1^), of halophyte shoot extracts (50 % ethanol). Means of three replicates followed by different letters are significantly different at *P *< 0.05.

The six halophytes studied here differed significantly in their iron-reducing power (Figure 2(Fig. 2)). *H. articulatum* shoots exhibited the highest reducing capacity, with the lowest EC_50_ value (380 µg**^.^**ml^-1^). *P. scoparius* and *D. cordata* exhibited moderate activity, whereas the other three halophytes were rather ineffective compared to the BHT standard (130 µg**^.^**ml^-1^).

### 2. Antimicrobial activity of halophyte shoot extracts

As shown above for their antioxidant capacity, the antimicrobial activity of the studied halophytes was much contrasted. *Haloxylon articulatum *shoot extract was found to possess the most powerful antibacterial activity, as it inhibited efficiently the growth of all the tested strains (Table 3(Tab. 3)). Noteworthy, the growth of *Salmonella enterica *and *Escherichia coli *was completely stopped and that of *Micrococcus luteus *was inhibited by 80 %*. *Moreover, *S. undulata*,* C. arabica*,* P. scoparius *and* C. colocynthis *exhibited a strong antibacterial activity, as they inhibited efficiently (by more than 50 %) the growth of three strains, particularly *S. aureus *(for *S. undulata* and *C. arabica*), *S. enterica* (for *P. scoparius*) and *E. coli *(for *C. colocynthis*). Finally, *D. cordata *extract was the less active halophyte, with only light effects on* E. coli*, *S. aureus* and *S. enterica *growth. Overall, bacterial strains may be classified on the basis of their sensitivity to halophyte extracts: *E. coli*, *S. aureus* and *S. enterica *were the most susceptible bacteria while *M. luteus *exhibited the strongest resistance toward halophytic extracts.

### 3. Tyrosinase inhibiting activities of halophyte shoot extracts

Inhibition of the two activities of tyrosinase, *i.e.* monophenolase (=cresolase) and diphenolase (=catechol oxidase), by halophyte shoot extracts was assessed through dopachrome formation. The obtained results showed significant (*P *< 0.05) differences in the inhibitory concentrations among the tested extracts (Table 4(Tab. 4)). The most active halophytes against monophenolase were *Cleome arabica* and *Pituranthos scoparius* (IC_50 _= 125 µg**^.^**ml^-1^), whereas the other four species were rather ineffective (IC_50_ > 1000 µg**^.^**ml^-1^). Conversely, every plant extract inhibited diphenolase activity, with *H. articulatum *exhibiting the strongest effect.

### 4. Aromatic composition of halophyte shoot extracts

Individual aromatic compounds were determined and assayed in ethanolic shoot extracts of the six halophytes. Nineteen compounds from different aromatic classes (amines, phenolic acids, flavonoids and diphenols) were identified and appeared to be diversely distributed in the studied plants (Table 5(Tab. 5)). Our results highlighted the richness of *Haloxylon articulatum*, and to a lesser extent *C. arabica*, in total aromatics. Thus, *H. articulatum* aromatic level (3.4 % DW) was 2.5 to 190 fold higher than that of the other species, *D. cordata *and* S. undulata* being particularly poor in aromatics.

Taking into consideration aromatic compound distribution, *C. colocynthis* and *S. undulata* displayed the highest diversity, with 6 molecules identified, while only four compounds were detected in *H. articulatum* shoots. Isorhamnetin-3*-o-*glucoside was found to be the major compound (1.6 mg**^.^**g^-1 ^DW) in *P. scoparius*, luteolin7-*o*-glucoside (7.7 mg**^.^**g^-1 ^DW) in* C. arabica*, and dopamine in *H. articulatum *and* C. colocynthis* (31.3 and 5 mg**^.^**g^-1 ^DW, respectively). On a qualitative basis, whereas phenolic acids and flavonoids were detected in every species, *H. articulatum *and *C. colocynthis* shoot extract were characterized by the presence of the aromatic amine dopamine. Besides, the former and *S. undulata* could be distinguished by the presence of the diphenol catechol and poor levels of flavonoids.

## Discussion

Since traditional ethnomedicinal use of plant is recognized as an important potential source for compounds used in mainstream medicine, six halophytic species widely used in African folk medicine have been selected for scientific investigations. Thus, antioxidant, antibacterial and anti-tyrosinase activities, as well as aromatic compound levels, were investigated in the selected species.

Antioxidant activities of the studied species differed greatly. Whatever the bioassay used (DPPH, *β*-carotene, superoxide, FRAP), *Haloxylon articulatum* showed the best antioxidant capacity as compared to the other halophytes. These results are in agreement with a recent report showing that *H. articulatum *possesses a strong ability to neutralize free radicals, as compared to other species including *Solenostemma oleifolium* (*Asclepiadaceae*) and *Echium pycnanthum* (*Boraginaceae*) (Chaouche et al., 2014[9]). Moreover, the antioxidant potential displayed by *H. articulatum* is the highest reported hitherto among *Chenopodiaceae* species (Hupel et al., 2011[22]; Magné, unpublished results). It is well known that phenolic compounds are powerful antioxidants in plant tissues. Accordingly, of the six halophytes studied, *H. articulatum* could be distinguished by its high level in gallic acid and catechol, two well-known strong antioxidants (Miura et al., 1998[33]; Kim et al., 2007[25]), and the adrenergic catechol derivative dopamine. The latter was found for the first time as the major compound in that species, thus could contribute, at least in part, to the strong antioxidant capacity of the halophyte (Yen and Hsieh, 1997[55]; Miura et al., 1998[33]). Besides, it is interesting to note that antioxidant capacity of each species depended on the test used. *Daemia cordata *showed a strong capacity to neutralize DPPH radical, as compared to the others species. These results are consistent with a recent report on *D. cordata* plants from other regions (Yakubu et al., 2015[54]). On this point, the halophyte *D. cordata* showed comparable antioxidant activity to other *Asclepiadaceae* species, for example *Solenostemma oleifolium* (Chaouche et al., 2014[9]). Besides, *Cleome Arabica *showed the best capacity to inhibit linoleic acid oxidation, as compared to the other species. As far as we know, this is the first study reporting such activity in *C. arabica*. It confirms that this species possesses strong antioxidant activities (Djeridane et al., 2010[13]). Finally, *Scorzonera undulata* was the most powerful quencher of superoxide anion. Literature on antioxidant properties of this species is scarce. Nevertheless, earlier study showed that it has an interesting antiradical activity with DPPH test (Harkati et al., 2013[20]). *Pituranthos scoparius*, although exhibiting a lower antioxidant activity than that of *H. articulatum*, showed higher antioxidant levels as compared to other *Apiaceae* species (Namjooyan et al., 2010[36]). Similar observations could be made with the Capparaceae *C. arabica* and the Asclepiadaceae *D. cordata*.

The antibacterial activities of shoot extracts were tested against four human pathogenic bacteria. The microplate bioassay results showed a significant variability in microbial growth inhibition depending on the plant species. As for the antioxidant activity, *H. articulatum *showed the best efficiency since it was the only halophyte inhibiting all tested bacterial strains. Earlier studies have supported that *H. articulatum *has an antibacterial activity against S*taphylococcus aureus *(Lamchouri et al., 2012[28]). With that respect, El-Shazly and Wink (2003[16]) showed that *H. articulatum *plants from Algeria are rich in antimicrobial molecules, including isoquinoline derivatives and β-carboline alkaloids. In our study, it could be that the strong antimicrobial activity of that species is the result of the abundance of phenolic compounds (Cueva et al., 2010[12]). In particular, such action might be due to dopamine since the aromatic amine and its derivatives have been reported to inhibit microbial growth (Pattan et al., 2009[39]; Maji et al., 2010[31]).

Besides, the other halophyte extracts inhibited strongly one bacterial strain, and more weakly two others. *Citrullus colocynthis* showed a significant inhibition of *E. coli*, in good agreement with a previous study using agar diffusion method on several Gram-bacteria (Bnyan et al., 2013[5]). Shoot extract of *Pituranthos scoparius* exhibited a strong antibacterial activity against *Salmonella enteritidis* (Gram-), confirming a recent report on *P. scoparius* plants from Algeria, toward *Salmonella typhimurium *growth (Houria et al., 2014[21]). *Scorzonera undulata *showed a strong inhibitory effect on S*taphylococcus aureus*, confirming a recent study using agar diffusion method on several S*taphylococcus *and* Pseudomomonas *strains (Kargol et al., 2013[23]). *Cleome arabica* caused a significant inhibition of Gram+ bacteria, especially *S. aureus*. This result, obtained for the first time with the accurate microdilution method, does not confirm earlier works which reported no antimicrobial activity of *C. arabica* plants from Algeria (Takhi et al., 2011[50]). At last, shoot extract of *D. cordata*, studied here for the first time, showed only a weak inhibitory effect against the tested strains (and no effect at all against *Micrococcus luteus*). The antibacterial activity is commonly related to extract composition. Interestingly, the most active halophyte against the studied bacteria accumulated the highest level of aromatic compounds. Accordingly, phenolic composition of the halophytes studied here (in particular phenolic acids, flavanones, flavanols and flavonols) could account for their antibacterial activities (Cowan, 1999[11]; Barber et al., 2000[3]).

It is well known that tyrosinase plays a critical role in catalyzing the melanogenic pathway since it promotes the production of reactive metabolites in the process of melanin formation (Sanchez-Ferrer et al., 1995[44]). The anti-tyrosinase effects were determined here for the first time in halophyte extracts, by assessing the hydroxylation of L-tyrosine to L-DOPA (monophenolase) and the oxidation of L-DOPA to DOPAquinone (diphenolase) according to the *in vitro* mushroom tyrosinase assay. Thus, *Cleome arabica* and *Pituranthos scoparius* extracts appeared as the sole active halophytes against monophenolase. Interestingly, the monophenolase inhibition capacity of these two species (IC_50_=125 µg**^.^**ml^-1^) was higher than that of many plants used for cosmetic purpose, particularly in skin whitening. These plants include *Erigeron annuus*, A*lbizzia julibrissin*, C*ornus macrophylla *and *Maackia floribunda *(Kim et al., 2007[25]). Besides, *H. articulatum *exhibited a strong inhibition of diphenolase activity*. *That capacity is close to those previously reported in several *Aloe *species, which are commonly used in skin-lightening preparations (Mapunya et al., 2012[32]). Overall, the differing inhibitory capacity of monophenolase and diphenolase activities observed within the same plant is likely due to the different mechanisms of tyrosinase inhibition. For example, as a catalyzer of oxidative reactions, tyrosinase may be inhibited by reducing agents such as phenolic compounds, which show a good affinity for the enzyme, thus reducing its catalytic capacity (Chang, 2009[8]). Alternatively, specific tyrosinase inactivators, such as mechanism-based inhibitors, form covalent bond with the enzyme, thus irreversibly inactivating the enzyme during catalytic reaction. They inhibit tyrosinase activity by inducing “suicide reaction”. Interestingly, Choi et al. (2008[10]) and Lee et al. (2009[29]) reported a positive correlation between antioxidant activity and tyrosinase inhibition. In our study, although no significant relationship could be found between anti-monophenolase and antioxidant activities, a positive correlation appeared between diphenolase inhibition and some antioxidant activities tested (*e.g.* FRAP or *β*-carotene bleaching inhibition) (Table 6(Tab. 6)). 

Such correlation may be explained by the fact that tyrosinase increases the oxidative burst in different physiological systems and may, therefore, be counteracted by antioxidant molecules (Sanchez-Ferrer et al., 1995[44]; Wang et al., 2011[53]). In order to explain the anti-tyrosinase activities of the studied halophytes, we investigated aromatic composition of each halophyte, and found a high level of aromatics in *H. articulatum* and, to a lesser extent, in *Cleome arabica*. Thus, the most aromatic-rich halophytes are also the most powerful diphenolase inhibitory species, which was confirmed by a strong positive correlation between total aromatic (TA) level and diphenolase inhibition (Table 6(Tab. 6)). To our knowledge, such relationship has never been reported hitherto. Furthermore, *H. articulatum* and *C. colocynthis* appeared to accumulate the aromatic amine dopamine. Since these two halophytes inhibit strongly diphenolase activity, but not monophenolase one, it could be that dopamine, being the result of DOPA decarboxylation, retro-inhibits dopachrome formation. Further experiments are under progress to ascertain that hypothesis, by investigating the *in vitro* effect of pure dopamine on both tyrosinase activities. Apart from this possible skin lightening action, a literature survey indicates that dopamine could be used as a valuable adrenergic, antimyocontractant, cardiotonic, diuretic, or hypertensive molecule, as well as to treat neurodegenerative troubles such as Alzheimer or Parkinson diseases (Duke, 1992[15]). Also, dopamine has been shown to possess antioxidant properties (Yen and Hsieh, 1997[55]). Therefore, *Haloxylon articulatum* could prove to be a valuable source of new drug against oxidative stress-associated syndromes.

## Conclusion

This study is the first to report simultaneously the antioxidant, antibacterial and anti-tyrosinase activities of six halophytes from Tunisian inland: *Citrullus colocynthis*, *Cleome arabica*, *Daemia cordata*,* Haloxylon articulatum*, *Pituranthos scoparius* and *Scorzonera undulata*. These species differ markedly in shoot antioxidant, antimicrobial, anti-tyrosinase activity, and their aromatic fingerprint was elucidated. Overall, our result highlights the strong potential of *H. articulatum *as a source of antioxidants, antimicrobials, as well as of whitening agents. With that respect, bioassay-guided fractionation of the ethanol extract of *H. articulatum* is under progress and will allow us to isolate the active compounds, making the extract or purified fractions promising sources of natural ingredients for cosmetic or food applications. Besides, the contribution of its principle compound dopamine to the biological activities described here will be elucidated.

## Acknowledgements

This work was supported by Campus France in the framework of the PHC CMCU program (n° 15G0812).

## Conflict of interest

The authors declare that they have no conflict of interest.

## Figures and Tables

**Table 1:**
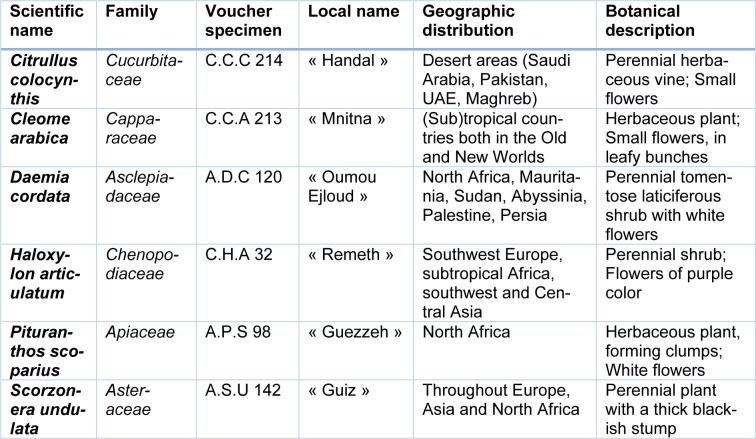
Characteristics of the six studied species

**Table 2:**
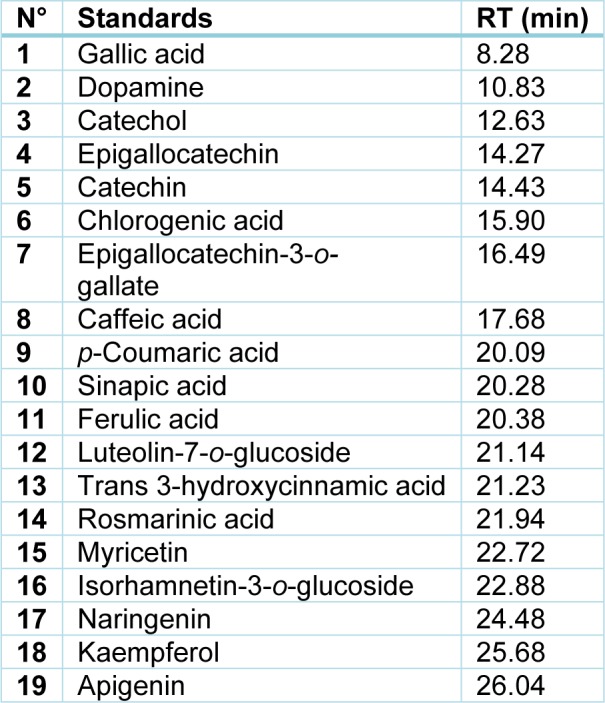
Retention times (RT) of aromatic standards determined by RP-HPLC (see 'Materials and Methods' section for details)

**Table 3:**
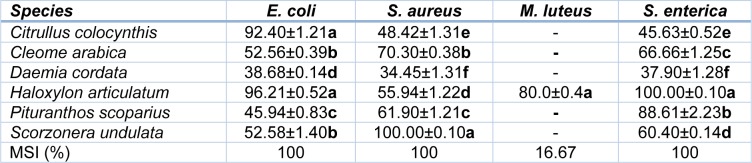
*In vitro* evaluation of antibacterial activity of halophyte extract against four pathogenic bacteria. Data are expressed as percent of growth inhibition induced by plant extract at the concentration of 1 mg^.^mL^-1^. Microbial Susceptibility Index (MSI) of microbe isolates (refer to text for details). In the same column, means ± SD of six replicates followed by different letters differ significantly at *P *< 0.05.

**Table 4:**
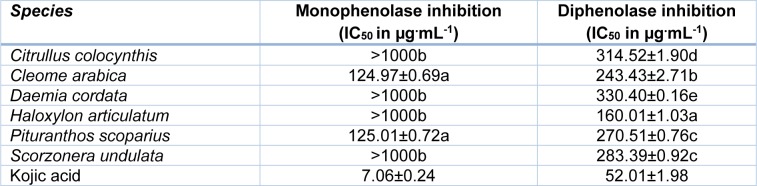
Anti-tyrosinase activities of halophyte shoot extracts (50 % ethanol). Activity against monophenolase and diphenolase were expressed as IC_50_ (µg^.^mL^-1^). In the same column, means ± SD of three replicates followed by different letters differ significantly at *P *< 0.05.

**Table 5:**
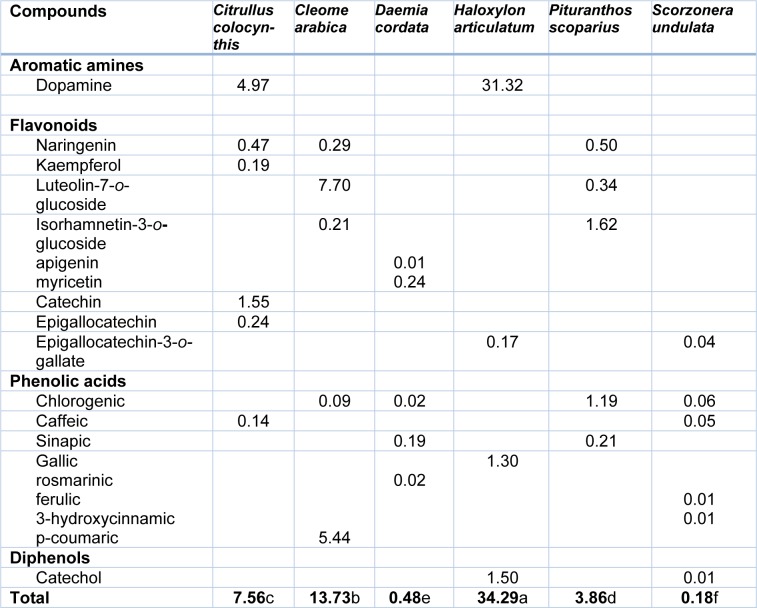
Aromatic compound distribution (mg^.^g^-1^DW) in shoot extracts of the studied halophytes, as analyzed by high-performance liquid chromatography (HPLC-DAD)

**Table 6:**
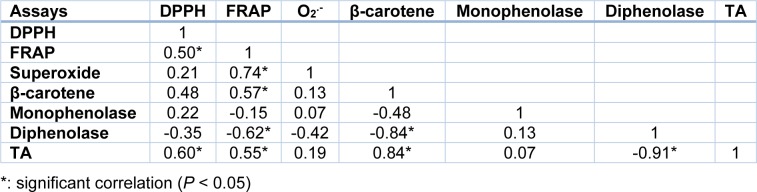
Correlation coefficients between total aromatic (TA) level, antioxidant and anti-tyrosinase (monophenolase and diphenolase inhibition) activities
